# A Discretization Approach for the Nonlinear Fractional Logistic Equation

**DOI:** 10.3390/e22111328

**Published:** 2020-11-21

**Authors:** Mohammad Izadi, Hari M. Srivastava

**Affiliations:** 1 Department of Applied Mathematics, Faculty of Mathematics and Computer, Shahid Bahonar University of Kerman, Kerman 76169-14111, Iran; 2 Department of Mathematics and Statistics, University of Victoria, Victoria, BC V8W 3R4, Canada; harimsri@math.uvic.ca; 3 Department of Medical Research, China Medical University Hospital, China Medical University, Taichung 40402, Taiwan; 4 Department of Mathematics and Informatics, Azerbaijan University, 71 Jeyhun Hajibeyli Street, Baku AZ1007, Azerbaijan

**Keywords:** logistic differential equation, liouville-caputo fractional derivative, local discontinuous galerkin methods, stability estimate

## Abstract

The present study aimed to develop and investigate the local discontinuous Galerkin method for the numerical solution of the fractional logistic differential equation, occurring in many biological and social science phenomena. The fractional derivative is described in the sense of Liouville-Caputo. Using the upwind numerical fluxes, the numerical stability of the method is proved in the $L_{\infty }$ norm. With the aid of the shifted Legendre polynomials, the weak form is reduced into a system of the algebraic equations to be solved in each subinterval. Furthermore, to handle the nonlinear term, the technique of product approximation is utilized. The utility of the present discretization technique and some well-known standard schemes is checked through numerical calculations on a range of linear and nonlinear problems with analytical solutions.

## 1. Introduction

In studies of elementary population dynamics the simplest model for the growth of a population is known as rate equation and structured by Malthus in (1798) [1]

(1) $$\left\{\begin{array}{cc} \frac{dM(t)}{dt} & =r\;M(t),\;t>0,\\ M(0) & =M_{0}, \end{array}\right.$$

where M(t) denotes the population at time *t*, the non-zero parameter *r* equals to r=β−α, where β and α are the per capita birth and death rates respectively. Here, $M_{0}$ is the population at time t=0. The exact analytical solution of Malthus population model (1) is explained the constant population growth rate $M\left(t\right)=M_{0}e^{rt}$. The Maithusian grow model is unrealistic over long times due to the fact that the solution of the rate equation is not included two main factors such as spread of diseases and the limitation on food supply. To model the effects of these factors in a population model, the logistic equation was considered by P. R. Verhulst in 1838 [2]

$$\frac{dN(t)}{dt}=rN\left(t\right)\left(1-\frac{N(t)}{K}\right),$$

where the variable $N\left(t\right)=M\left(t\right)/M_{max}$ is the whole population and normalized to its maximum attainable value $M_{max}$, *r* denotes the intrinsic growth rate while the constant K>0 known as the carrying capacity of the environment. By defining X(t):=N(t)/K and σ:=rK, the standard logistic equation can be rewritten as

(2) $$\left\{\begin{array}{cc} \frac{dX(t)}{dt} & =\sigma \;X\left(t\right)\;\left(1-X(t)\right),\;t>0,\\ X(0) & =X_{0}. \end{array}\right.$$

where $X_{0}=M\left(0\right)/M_{max}$. The exact solution of this equation can be easily obtained as

$$X\left(t\right)=\frac{X_{0}}{X_{0}+\left(1-X_{0}\right)e^{-\sigma t}}.$$

In the last decades, many efforts have been devoted to extend the integer-order models to the corresponding fractional-order models, which are more descriptive and can provide a powerful and valuable instrument for the explanation of hereditary and memory properties of several materials and process [3,4]. Replacing the classical derivative operator in (2) by a fractional one, the fractional logistic equation will be obtained. This model of population growth has been found applications in numerous disciplines of science and engineering. For instance, the growth of tumors in medicine [5] can be modelled as the fractional logistic equation (FLE). In addition, the milstone of various important mathematical models is based on the fractional logistic equation such as two models in Radar signals [6] and electroanalytical chemistry [7]. Several variations of the population growth model have been studied in the literature [8]. In the present study, we are going to investigate the following logistic population model of fractional order in the form

(3) $$\left\{\begin{array}{cc} \; & {}_{a}^{\mathrm{LC}}D_{t}^{\nu }X\left(t\right)=\sigma \;X\left(t\right)\;\left(1-X(t)\right)=:\sigma \;X\left(t\right)\;g\left(X\left(t\right)\right),\;t>0,\\ \; & X\left(0\right)=X_{0}, \end{array}\right.$$

where the symbol ${}_{a}^{\mathrm{LC}}D_{t}^{\nu }$ denotes the fractional derivative operator of Liouville-Caputo type and ν∈(0,1]. It should be emphasize that in (3) we have used the function g(s)≡1−s, which corresponds to the nonlinear logistic equation. However, to address the linear counterpart of this equation we also consider g(s)≡1. The issue of existence and the uniqueness of the solution of (3) is discussed in details in Reference [9].

It is known that for most fractional differential equations there is no possibility to find the exact solutions analytically. Consequently, exploring an approximate or numerical technique is of primary interest for such fractional equations. Many efforts have been made toward the exact analytical solution of the problem (3). The first one is proposed by West [10], which is based on the Carleman embedding technique. Later, it is shown that in Reference [11] the this analytical function is only very close to the numerical solutions of the FLE. The other analytical methods for the FLE include the fractional Taylor expansion method [12], a method based on Euler’s numbers [13], and the varational iterative method [14]. Besides the analytical investigations, numerous computational approaches have been proposed for the nonlinear FLE. Let us mention the predictor-corrector approaches [9,15], the finite difference schemes [14,16], the spectral methods [17,18], the Bessel collocation method [19], the Chebyshev wavelet method [20], the Laguerre collocation method [21], and the fractional spline collocation method [22].

Many other numerical and approximation methods as well as computational approaches have been developed and applied for the FDEs which are based upon various closely-related models of real-world problems. For example, Baleanu et al. [23] made use of a Chebyshev spectral method based on operational matrices, a remarkable survey of numerical methods can be found in [24], a study of the fractional-order Bessel, Chelyshkov, and Legendre collocation schemes for the fractional Riccati equation was presented in [25], an operational matrix of fractional-order derivatives of Fibonacci polynomials was developed in [26], an introductory overview and recent developments involving FDEs was presented in [27], efficiency of the spectral collocation method in the dynamic simulation of the fractional-order epidemiological model of the Ebola virus was investigated in [28], the Jacobi collocation method and a spectral tau method based on shifted second-kind Chebyshev polynomilas for the approximate solution of some families of the fractional-order Riccati differential equations were discussed in [29,30], computational approaches to FDEs for the biological population model were discussed in [31], the generalized Chebyshev and Bessel colllocation approaches for fractional BVPs and multi-order FDEs were considered in [32,33], and a general wavelet quasi-linearization method for solving fractional-order population growth model was developed and applied in [34].

In this work, we take a further step towards proposing a numerical method for solving the FLE. We utilize a discontinuous finite element approach, i.e, the local discontinuous Galerkin (LDG) discretization approach for the FLE (3). To apply the LDG scheme, we must rewrite a given FDEs as a system of first-order ordinary differential equations (ODEs) with together a fractional integral. Hence, the discontinuous Galerkin (DG) method is employed to discretize the resulting system as well as the fractional integral. The first DG method was introduced by Reed and Hill [35] in 1973 for numerically solving neutron transport, that is, a time-independent linear hyperbolic equation. Since then the DG schemes have been well implemented for the classical ODEs was started by the work [36]. DG schemes as a subclass of finite element methods (FEMs) allow us to exploit discontinuous discrete basis functions. These local basis functions are usually selected as piecewise polynomials. Exploiting completely discontinuous basis functions offers great opportunities compared to traditional FEMs when used to discretize differential equations. In summary, the main gains of the DG methods are in terms of flexibility, accuracy as well as parallelizability, see cf. Reference [37].

To the best of our knowledge, the LDG approaches for the ODEs of fractiona-order including one-term and multi-terms were first discussed in Reference [38] and then have been applied to many model problems [39,40,41]. It is worth mentioning that the success of LDG methods is based on the designing of appropriate numerical fluxes at the interface elements. In this work, we utilize the upwind numerical flux as natural choice for the FLE. By choosing the upwind fluxes we arel able to prove the numerical stability of the LDG scheme.

The rest of this paper is organized as follows. In the next Section, we review some fractional calculus preliminaries and state some of their properties that will be used later on. The formulation of the LDG scheme for the logistic equation is established in Section 3. Hence, the algebraic form of the LDG scheme is obtained with the aid of shifted Legendre basis functions. The technique of product approximation is also applied to deal with the nonlinear term in the weak formulation. In Section 4 we establish the numerical stability of the scheme in the linear case and a discussion about the error estimation is made. In Section 5, the applicability and utility of the present numerical schemes are verified by performing several simulations on two linear and nonlinear population growth and logistic model problems. Finally, a conclusion is drawn in Section 6.

## 2. Fractional Calculus

Now, we present some fundamental and mathematical preliminaries of the fractional calculus theory to be utilized in our subsequent sections, see References [3,4,27].

**Definition** **1.** *Let ν≥0 is given. The (left) Riemann-Liouville fractional integral operator of order ν is given by*

$$I^{\nu }f\left(t\right)\equiv {}_{a}I_{t}^{\nu }f\left(t\right)=\left\{\begin{array}{cc} \frac{1}{\Gamma (\nu )}\int_{a}^{t}f\left(p\right)\;{\left(t-p\right)}^{\nu -1}\;dp,\; & \nu >0,\;t>0,\\ f(t),\; & \nu =0. \end{array}\right.$$

The integral operator $I^{\nu }$ has many properties. Among others, we make use of the followings

(1) $I^{\nu }I^{\beta }f\left(t\right)=I^{\nu +\beta }f\left(t\right),$
(2) $I^{\nu }\left(c_{1}f\left(t\right)+c_{2}g\left(t\right)\right)=c_{1}I^{\nu }f\left(t\right)+c_{2}I^{\nu }g\left(t\right),\;c_{1},c_{2}\in R,$
(3) $I^{\nu }t^{\gamma }=\frac{\Gamma (\gamma +1)}{\Gamma (\gamma +\nu +1)}t^{\nu +\gamma },\;\gamma >-1.$

The corresponding definition of the right Riemann-Liouville fractional integral on the interval [t,b] instead of [a,t] is given by

$${}_{t}I_{b}^{\nu }f\left(t\right)=\frac{1}{\Gamma (\nu )}\int_{t}^{b}f\left(p\right)\;{\left(p-t\right)}^{\nu -1}\;dp,\;\nu >0,\;t>0.$$

**Definition** **2.** *The fractional derivative $D^{\nu }$ of f(t) in the Liouville-Caputo’s sense is defined as*

$$D^{\nu }f\left(t\right)\equiv {}_{a}^{\mathrm{LC}}D_{t}^{\nu }f\left(t\right)=\left\{\begin{array}{cc} \frac{1}{\Gamma (m-\nu )}\int_{a}^{t}\frac{f^{\left(m\right)}\left(p\right)}{{\left(t-p\right)}^{\nu -m+1}}\;dp,\; & m-1<\nu <m,\;t>0,\\ f^{\left(m\right)}\left(t\right),\; & \nu =m,\;m\in N. \end{array}\right.$$

We make use of the following [4]:

(4) $$\begin{array}{cc} \; & D^{\nu }\left(C\right)=0\;\left(C\;\mathrm{is a constantq}\right), \end{array}$$

(5) $$\begin{array}{cc} \; & D^{\nu }\;t^{\gamma }=\left\{\begin{array}{cc} \frac{\Gamma (\gamma +1)}{\Gamma (\gamma +1-\nu )}t^{\gamma -\nu }, & \mathrm{for}\;\gamma \in N_{0}\;\mathrm{and}\;\gamma \geq \left\lceil \nu \right\rceil ,\;\mathrm{or}\;\gamma \notin N_{0}\;\mathrm{and}\;\gamma >\left\lfloor \nu \right\rfloor ,\\ 0, & \mathrm{for}\;\gamma \in N_{0}\;\mathrm{and}\;\gamma <\left\lceil \nu \right\rceil . \end{array}\right. \end{array}$$

Here, we have used the ceiling and floor functions ⌈ν⌉, ⌊ν⌋ respectively. It should be noted that, two operators $I^{\nu }$ and $D^{\nu }$ are related through the following expression

(6) $$D^{\nu }f\left(t\right)=I^{m-\nu }D^{m}f\left(t\right),\;D=\frac{d}{dt}.$$

## 3. Discretized LDG Formulation

In order to formulate the LDG method for the logistic equation in (3), some basic notations will first be introduced.

Let us consider (3) on L=(0,T) for some given T>0. To rewrite (3) as a first-order system, we introduce two new variables $z_{0}\left(t\right)=X\left(t\right)$ and $z_{1}\left(t\right)=\frac{dX(t)}{dt}$ and use the relation (6) to get

(7) $$\left\{\begin{array}{cc} \; & z_{1}\left(t\right)-\frac{dz_{0}\left(t\right)}{dt}=0,\\ \; & {}_{0}I_{t}^{\left(1-\nu \right)}z_{1}\left(t\right)-\sigma \;z_{0}\left(t\right)\;\left(1-z_{0}\left(t\right)\right)=0,\\ \; & z_{0}\left(0\right)-X_{0}=0, \end{array}\right.$$

being ν∈(0,1] and t∈L. By Δ we denote a partitioning of the interval *L* into into *J* subintervals $L_{l}=\left(t_{l-1},t_{l}\right)$ for l=1,…,J. The grid points of Δ will be denoted as

$$0=:t_{0}<t_{1}<\ldots <t_{J-1}<t_{J}:=T.$$

By $h_{l}$ we mean the length of each $L_{l}$, that is, $h_{l}=t_{l}-t_{l-1}$ for l=1,2,…,N. The maximum length of these element is taken as $h:=\max_{l=1}^{J}h_{l}$. We associate the mesh Δ with the broken Sobolev spaces

$$H_{\Delta }^{1}=\left\{w:L\to R\;|\;w{|}_{L_{l}}\in H^{1}\left(L_{l}\right),\;l=1,2,\ldots ,J\right\}.$$

and

$$S_{\Delta }=\left\{w:L\to R\;|\;w{|}_{L_{l}}\in L_{2}\left(L_{l}\right),\;l=1,2,\ldots ,J\right\},$$

By using these function spaces, let assume that the solutions of system (7) belong to corresponding spaces

$$\left(z_{0}\left(t\right),z_{1}\left(t\right)\right)\in H_{\Delta }^{1}\times S_{\Delta }.$$

It should be noted that the elements of space $H_{\Delta }^{1}$ may be discontinuous in *t* at discrete time level $t_{1}$. In this respect, at the mesh grid points, defining the left-sided as well as the right-sided limits of a function *w* is necessary, where w:L→R is a piecewise continuous function. By $w_{n}^{-}$ and $w_{n}^{+}$, we let the left- and right-sided limits of *w* at $t_{l}$

$$w_{l}^{+}=w^{+}\left(t_{l}\right)=w\left(t_{l}^{+}\right):=\lim_{s\to 0^{+}}w\left(t_{n}+s\right),\;w_{l}^{-}=w^{-}\left(t_{l}\right)=w\left(t_{l}^{-}\right):=\lim_{t\to 0^{-}}w\left(t_{n}+s\right).$$

For any positive integer number *r*, we denote by $P_{r}\left(L_{l}\right)$ the space of polynomials of degree less or equal than *r* on the element $L_{l}\in \Delta$. We then let the approximate solutions $z_{0}\left(t\right),z_{1}\left(t\right)$ belong to a subspace $V^{\left(r\right)}\subset H_{\Delta }^{1}$, which is a finite dimensional space. This subspace is defined as the space of discontinuous and piecewise polynomial functions

$$V^{\left(r\right)}=\left\{w:L\to R\;|\;w{|}_{L_{l}}\in P_{r}\left(L_{l}\right),\;l=1,2,\ldots ,J\right\}.$$

We further define $Z_{0}\left(t\right)$ and $Z_{1}\left(t\right)$ as the DG approximations to the exact solutions $z_{0}\left(t\right)$ and $z_{1}\left(t\right)$ of the system (7) respectively on the element $L_{l}$. Below, we make use of the following notations

$${\left(w,\;v\right)}_{l}:=\int_{L_{l}}w\;v\;dt,\;{\langle w,\;v\rangle }_{l}:=\int_{0}^{t_{l}}w\;v\;dt,\;{∥w∥}_{l}:=\sqrt{{\langle w,\;w\rangle }_{l}}.$$

For obtaining the weak DG formulation, we first multiply the first equation in (7) by a test function $w_{0}\in V^{\left(r\right)}$ and integrate over $L_{l}$. By applying the integrating by parts we get

(8) $${\left(Z_{1}\left(t\right),\;w_{0}\right)}_{l}+{\left(Z_{0}\left(t\right),\;\frac{dw_{0}}{dt}\right)}_{l}-Z_{0}\left(t_{l}^{-}\right)\;w_{0}\left(t_{l}^{-}\right)+Z_{0}\left(t_{l-1}^{+}\right)\;w_{0}\left(t_{l-1}^{+}\right)=0.$$

Hence, the second integral equation in (7) will multiplied by a test function $w_{1}\in V^{\left(r\right)}$ and integrate over $L_{l}$. To advance the solution in time, we replace $Z_{0}\left(t_{l-1}^{+}\right)$ by the upwind flux $Z_{0}\left(t_{l-1}^{-}\right)$ in (8). Thus, the discrete formulation for finding $Z_{0},Z_{1}\in V^{\left(r\right)}$ takes the following form for all $w_{0},w_{1}\in V^{\left(r\right)}$, and l=1,2,…,J

(9) $$\left\{\begin{array}{c} {\left(Z_{1}\left(t\right),\;w_{0}\left(t\right)\right)}_{l}+{\left(Z_{0}\left(t\right),\;w_{0}^{\prime }\left(t\right)\right)}_{l}-Z_{0}\left(t_{l}^{-}\right)\;w_{0}\left(t_{l}^{-}\right)+Z_{0}\left(t_{l-1}^{-}\right)\;w_{0}\left(t_{l-1}^{+}\right)=0,\\ {\left({}_{0}I_{t}^{\left(1-\nu \right)}Z_{1}\left(t\right),\;w_{1}\left(t\right)\right)}_{l}-\sigma \;{\left(Z_{0}\left(t\right),\;w_{1}\left(t\right)\right)}_{l}+\sigma \;{\left(Z_{0}^{2}\left(t\right),\;w_{1}\left(t\right)\right)}_{l}=0,\\ Z_{0}\left(t_{0}^{-}=0\right)-X_{0}=0. \end{array}\right.$$

It should be noted that, to start computations on the first element $L_{1}=\left(t_{0},t_{1}\right)$ we use the given initial condition $Z_{0}\left(t_{0}^{-}\right)=X_{0}$. Hence, by utilizing the upwind flux as natural choice, we are able to solve the resultant equations element by element on each subinterval $L_{l}$ for l=1,2,…,J. In each element, we just need to invert a local matrix of size (r+1)×(r+1) in place of a global matrix of size J(r+1)×J(r+1).

### Algebraic Formulation

Since the functions in $V^{\left(r\right)}$ may be discontinuous across interfaces of the element, various local bases can be selected for finite element approximation in (9). Let us choose a basis in the space $P_{r}\left(L_{l}\right)$ formed by functions $\phi_{0}^{l},\phi_{1}^{l},\ldots ,\phi_{r}^{l}$. Thus the numerical approximations $Z_{0}$ of $z_{0}$ and $Z_{1}$ of $z_{1}$ in every element $L_{l}$ can be expressed as

(10) $$Z_{0}\left(t\right)=\sum_{i=0}^{q}\alpha_{i}^{l}\;\phi_{i}^{l}\left(t\right),\;Z_{1}\left(t\right)=\sum_{i=0}^{q}\beta_{i}^{l}\;\phi_{i}^{l}\left(t\right),\;t\in L_{l}.$$

Here, the coefficients $\alpha_{i}^{l},\beta_{i}^{l},i=0,\ldots ,r$ denote the degrees of freedom to be sought in each $L_{l}$,l=1,…,J. To proceed, we take the test functions in each element $L_{l}$ in the form $w_{j}=\phi_{j}^{l}\left(t\right)$ for j=0,1,…,r and l=0,1,…,J. Now, by specifying the basis functions as we done below, the discrete LDG formulation (9) is reduced to a algebraic system of equations.

For practical implementation of the LDG scheme (9) for the FLE (3), we use the set of orthogonal Legendre polynomials for the space $V^{\left(r\right)}$. Let us recall that, the *i*’th degree Legendre polynomials $P_{i}\left(s\right)$ can be generated by the well-known Rodriguez formula

$$P_{i}\left(s\right)=\frac{1}{2^{i}i!}\frac{d^{i}}{ds^{i}}{\left(s^{2}-1\right)}^{i}.$$

The Legendre polynomials satisfy the following relations [17]

(11a) $$\begin{array}{cc} \; & \int_{-1}^{1}P_{i}\left(s\right)\;P_{j}\left(s\right)ds=\frac{2\delta_{ij}}{2i+1},\;P_{i}\left(1\right)=1,\;P_{i}\left(s\right)={\left(-1\right)}^{i}P_{i}\left(-s\right),\;i,j\geq 0, \end{array}$$

(11b) $$\begin{array}{cc} \; & \left(2i+1\right)P_{i}\left(s\right)=\frac{dP_{i+1}\left(s\right)}{ds}-\frac{dP_{i-1}\left(s\right)}{ds}, \end{array}$$

where $\delta_{ij}$ denotes the Kronecker delta. The first property shows that these set of orthogonal polynomials are orthogonal with respect to weighting function w(t)≡1 on (−1,1). The Legendre polynomial $P_{i}\left(s\right)$ of degree *i* can be explicitly expressed as follows

Pi(s)=∑k=0Miciksi−2k,cik:=12i(−1)kik2i−2ki,

where $M_{i}=i/2$ or (i−1)/2, whichever is an integer. Due to the fact that these polynomials are orthogonal on [−1,1], we map them onto the element $L_{l}$ by using the following change of variable

$$s:=\frac{2t-t_{l-1}-t_{l}}{h_{l}},\;t\in L_{l}.$$

Let the resultant shifted Legendre polynomials denoted by $L_{i}\left(t\right)$. Thus, the explicit form of $L_{i}\left(t\right)$ of degree *i* takes the form

$$L_{i}\left(t\right)=\sum_{k=0}^{M_{i}}c_{ik}\;{\left(\frac{2t-t_{l-1}-t_{l}}{h_{l}}\right)}^{i-2k}.$$

By means of the binomial formula, one can further simplify the last expression as follows

(12) $$L_{i}\left(t\right)=\sum_{k=0}^{M_{i}}\sum_{m=0}^{i-2k}C_{ikm}\;t^{m},$$

where the coefficients $C_{ikm}$ are defined as

$$C_{ikm}:=\frac{{\left(-1\right)}^{i+k+m}\;\left(2i-2k\right)!}{2^{i}\;\left(i-k\right)!\;k!\;l!\;\left(i-2k-m\right)!}{\left(\frac{t_{l}+t_{l-1}}{t_{l}-t_{l-1}}\right)}^{i-2k}{\left(\frac{2}{t_{l}+t_{l-1}}\right)}^{m}.$$

Now, we choose $\phi_{i}^{l}\left(t\right)=L_{i}\left(t\right)$ in (10) for l=1,2,…,J, where $L_{i}$ is the shifted Legendre polynomial of degree *i* in *t* defined in (12). With this transformation, the unknown values $\alpha_{i}^{l},\beta_{i}^{l}$ in (10) can be interpreted as the Legendre coefficients of the expansion of $Z_{0},Z_{1}$. Hence, by the virtue of the Legendre properties (11) and inserting (10) into the discrete formulation (9) we have for l=1,…,J as

(13) $$\begin{array}{cc} \; & \sum_{i=0}^{r}\beta_{i}^{l}{\left(L_{i}\left(t\right),\;L_{j}\left(t\right)\right)}_{l}+\sum_{i=0}^{r}\alpha_{i}^{l}{\left(L_{i}\left(t\right),\;L_{j}^{\prime }\left(t\right)\right)}_{l}-\sum_{i=0}^{r}\alpha_{i}^{l}+\sum_{i=0}^{r}\alpha_{i}^{l-1}{\left(-1\right)}^{j}=0,\\ \; & \sum_{i=0}^{r}\beta_{i}^{l}{\left({}_{0}I_{t}^{\left(1-\nu \right)}L_{i}\left(t\right),\;L_{j}\left(t\right)\right)}_{l}-\sigma \sum_{i=0}^{r}\alpha_{i}^{l}{\left(L_{i}\left(t\right),\;L_{j}\left(t\right)\right)}_{l}+\sigma {\left({\left[\sum_{i=0}^{r}\alpha_{i}^{l}\;L_{i}\left(t\right)\right]}^{2},\;L_{j}\left(t\right)\right)}_{l}=0, \end{array}$$

for j=0,…,r. To proceed, we need to deal with two main difficulties involving the integral and nonlinear terms that appear in (13). To tackle the integral term, the properties (1)–(3) of fractional integration in Section 2 is used to obtain

$${}_{0}I_{t}^{\left(1-\nu \right)}L_{i}\left(t\right)=\sum_{k=0}^{M_{i}}\sum_{m=0}^{i-2k}C_{ikm}\;{}_{0}I_{t}^{\left(1-\nu \right)}t^{m}=\sum_{k=0}^{M_{i}}\sum_{m=0}^{i-2k}C_{ikm}^{\prime }\;t^{m+1-\nu },\;C_{ikm}^{\prime }:=C_{ikm}\;\frac{\Gamma (m+1)}{\Gamma (m+2-\nu )}.$$

Next, the explicit form (12) is utilized for $L_{j}\left(t\right)$ and then ${}_{0}I_{t}^{\left(1-\nu \right)}L_{i}\left(t\right)$ will be inserted into the inner product. Now, by integration over $L_{l}$ we obtain

(14) $$d_{i,j}:={\left({}_{0}I_{t}^{\left(1-\nu \right)}L_{i}\left(t\right),\;L_{j}\left(t\right)\right)}_{l}=\sum_{k=0}^{M_{i}}\sum_{m=0}^{i-2k}\sum_{k^{\prime }=0}^{M_{j}}\sum_{m^{\prime }=0}^{j-2k^{\prime }}C_{ikmjk^{\prime }m^{\prime }}^{\prime \prime }\left(t_{l}^{m+m^{\prime }+2-\nu }-t_{l-1}^{m+m^{\prime }+2-\nu }\right),$$

with the coefficients

$$C_{ikmjk^{\prime }m^{\prime }}^{\prime \prime }:=C_{ikm}^{\prime }\;C_{jk^{\prime }m^{\prime }}/\left(m+m^{\prime }+2-\nu \right).$$

Th nonlinear term in (13) can be computed using the Legendre polynomials. For instance, if r=1 we may write it as a product of two vectors

$$n_{j}^{DC}:={\left(Z_{0}^{2}\left(t\right),\;L_{j}\left(t\right)\right)}_{l}=\left[{\left[\alpha_{0}^{l}\right]}^{2},2\alpha_{0}^{l}\alpha_{1}^{l},{\left[\alpha_{1}^{l}\right]}^{2}\right]\;\cdot \;\int_{L_{l}}{\left[L_{0}^{2}\left(t\right),L_{0}\left(t\right)L_{1}\left(t\right),L_{1}^{2}\left(t\right)\right]}^{T}L_{j}\left(t\right)dt,$$

for j=0,1. Therefore, it is not a difficult task to calculate $n_{j}^{DC}$ by direct computation (D.C.) using the shifted Legendre polynomials on each $L_{l}$ for different *j*. Of course one may exploit the symbolic toolbox in Matlab to facilitate the process of integration of these polynomials. Alternatively, to handle the nonlinear term in (13), the product approximation (P.A.) technique [42] is used in the following manner

$$Z_{0}^{2}\left(t\right)={\left[\sum_{i=0}^{r}\alpha_{i}^{l}\;L_{i}\left(t\right)\right]}^{2}\approx \sum_{i=0}^{r}{\left[\alpha_{i}^{l}\right]}^{2}\;L_{i}\left(t\right).$$

This technique enables us to write the nonlinear part as

(15) $$n_{i,j}^{PA}:={\left(Z_{0}^{2}\left(t\right),\;L_{j}\left(t\right)\right)}_{l}=\sum_{i=0}^{r}{\left[\alpha_{i}^{l}\right]}^{2}\;{\left(L_{i}\left(t\right),\;L_{j}\left(t\right)\right)}_{l}.$$

Now, it suffices to calculate the two first terms in (13). To this end, we compute the elements of the mass matrix as

(16) $$m_{i,j}:={\left(L_{i}\left(t\right),\;L_{j}\left(t\right)\right)}_{l}=\int_{L_{l}}L_{i}\left(t\right)L_{j}\left(t\right)dt=\left\{\begin{array}{cc} \frac{h_{l}}{2i+1},\; & i=j,\\ 0,\; & i\neq j. \end{array}\right.$$

Finally, the entries of the stiffness matrix

$$s_{i,j}={\left(L_{i}\left(t\right),\;L_{j}^{\prime }\left(t\right)\right)}_{l}=\int_{L_{l}}L_{i}\left(t\right)L_{j}^{\prime }\left(t\right)dt.$$

need to be calculated. In the new coordinate, we recursively employ the Legendre property (11b) to derive

$$\frac{h_{l}}{2}L_{i+1}^{\prime }\left(t\right)=\left(2i+1\right)L_{i}\left(t\right)+\left(2\left(i-1\right)+1\right)L_{i-2}\left(t\right)+\left(2\left(i-4\right)+1\right)L_{i-4}\left(t\right)+\cdots .$$

By applying the orthogonality relation (11a) to the preceding equation and then simplifying the involved integral in $s_{i,j}$, we finally get

(17) $$s_{i,j}=\left\{\begin{array}{cc} 2, & \mathrm{if}\;i>j\;\mathrm{and}\;(i+j)\;\mathrm{is even},\\ 0, & \mathrm{otherwise}. \end{array}\right.$$

Using (14)–(17), one may write (13) in the matrix-vector multiplication form for l=1,…,J as follows

(18) $$\left\{\begin{array}{cc} \; & M\beta^{l}+\left(S-E\right)\alpha^{l}=b^{l},\\ \; & D\beta^{l}-\sigma M\left(\alpha^{l}-\alpha^{2,l}\right)=0, \end{array}\right.$$

where the unknown vectors $\alpha^{l},\beta^{l}$, and $\alpha^{2,l}$ are defined

$$\alpha^{l}={\left(\begin{array}{c} \alpha_{0}^{l},\ldots ,\alpha_{r}^{l} \end{array}\right)}^{T},\beta^{l}={\left(\begin{array}{c} \beta_{0}^{l},\ldots ,\beta_{r}^{l} \end{array}\right)}^{T},\alpha^{2,l}={\left(\begin{array}{c} {\left[\alpha_{0}^{l}\right]}^{2},\ldots ,{\left[\alpha_{r}^{l}\right]}^{2} \end{array}\right)}^{T}.$$

Note in (18) that the components of matrix E are $e_{i,j}:=1$ while that of M,S,N and D are $m_{i,j},s_{i,j},n_{i,j},$ and $d_{i,j}$ respectively for i,j=0,…,r as defined above. Moreover, the components of the known vector $b^{l}$ are

$$b_{i}:={\left(-1\right)}^{i+1}Z_{0}\left(t_{l-1}^{-}\right),\;i=0,1,\ldots ,r.$$

Clearly, the value of $Z_{0}\left(t_{l-1}^{-}\right)$ is already known from the preceding time interval $L_{l-1}$. Obviously this value at the first time interval is computed as $X_{0}$, which known as the initial condition. Also, the obtained system (18) is a nonlinear algebraic system of equations have to be solved in each $L_{l}$ for l=1,…,J. This system can be solved for example, via Newton type schemes. It is known that this method converges quadratically whenever the approximation is close to the actual solution of the given nonlinear system. Using the D.C. approach, we also arrive at a nonlinear system of equation in the general form $F(\alpha^{l},\beta^{l})=0$ to be solved in each interval $L_{l}$. As we show in the numerical experiments, this approach is more accurate than the corresponding P.A. approach.

## 4. Numerical Stability and Error Estimates

Now, we are going to establish the stability of proposed LDG scheme when applied to the logistic equation in the linear case by considering g(t)≡1 in (3). In this case we have

(19) $$\left\{\begin{array}{cc} \; & {}_{a}^{\mathrm{LC}}D_{t}^{\nu }X\left(t\right)=\sigma \;X\left(t\right),\;\nu \in \left(0,1\right).\\ \; & X\left(0\right)=X_{0}. \end{array}\right.$$

Without loss of generality, let us assume that σ<0. The numerical scheme of (19) is to find $Z_{0},Z_{1}\in V^{\left(r\right)}$ such that

(20) $$\left\{\begin{array}{cc} \; & Z_{0}\left(t_{l}^{-}\right)\;w_{0}\left(t_{l}^{-}\right)-Z_{0}\left(t_{l-1}^{-}\right)\;w_{0}\left(t_{l-1}^{+}\right)-{\left(Z_{1}\left(t\right),\;w_{0}\left(t\right)\right)}_{l}-{\left(Z_{0}\left(t\right),\;w_{0}^{\prime }\left(t\right)\right)}_{l}=0,\\ \; & {\left({}_{0}I_{t}^{\left(1-\nu \right)}Z_{1}\left(t\right),\;w_{1}\left(t\right)\right)}_{l}=\sigma \;{\left(Z_{0}\left(t\right),\;w_{1}\left(t\right)\right)}_{l},\\ \; & Z_{0}\left(t_{0}^{-}\right)-X_{0}=0, \end{array}\right.$$

for all $w_{0},w_{1}\in V^{\left(r\right)}$, and l=1,2,…,J. Let us state the next lemma, which based on the semigroup properties of fractional integral operators and will be used below, a proof of which can be found in Reference [38].

**Lemma** **1.** Suppose that ν∈(0,1), then we have

$$\begin{array}{cc} {\langle {}_{0}I_{t}^{1-\nu }u,\;u\rangle }_{l} & ={\langle {}_{0}I_{t}^{\frac{1-\nu }{2}}u,\;{}_{t}I_{t_{l}}^{\frac{1-\nu }{2}}u\rangle }_{l}=\cos\left(\frac{(1-\nu )\pi }{2}\right){∥u∥}_{H^{\frac{1-\nu }{2}}\left(\left[0,t_{l}\right]\right)}^{2}. \end{array}$$

Let us assume that ${\tilde{Z}}_{0},{\tilde{Z}}_{1}\in V^{\left(r\right)}$ be the approximate solutions of $Z_{0},Z_{1}$ respectively. Now, the numerical errors are defined as $E_{X_{i}}:={\tilde{Z}}_{i}-Z_{i}$ for i=0,1. It can be seen that ${\tilde{Z}}_{0}$ and ${\tilde{Z}}_{1}$ both satisfy (20). If we subtract Equation (20) from the same equations with ${\tilde{Z}}_{0}$ and ${\tilde{Z}}_{1}$, the following error equations will be obtained

(21) $$\left\{\begin{array}{cc} \; & E_{X_{0}}\left(t_{l}^{-}\right)\;w_{0}\left(t_{l}^{-}\right)-E_{X_{0}}\left(t_{l-1}^{-}\right)\;w_{0}\left(t_{l-1}^{+}\right)-{\left(E_{X_{1}}\left(t\right),\;w_{0}\left(t\right)\right)}_{l}-{\left(E_{X_{0}}\left(t\right),\;w_{0}^{\prime }\left(t\right)\right)}_{l}=0,\\ \; & -\frac{1}{\sigma }\;{\left({}_{0}I_{t}^{\left(1-\nu \right)}E_{X_{1}}\left(t\right),\;w_{1}\left(t\right)\right)}_{l}=-{\left(E_{X_{0}}\left(t\right),\;w_{1}\left(t\right)\right)}_{l}, \end{array}\right.$$

which holds for all $w_{0},w_{1}\in V^{\left(r\right)}$. Taking $w_{0}=E_{X_{0}}$ and $w_{1}=E_{X_{1}}$ in (21) followed by collecting these two equations, we conclude that

$$\begin{array}{c} E_{X_{0}}^{2}\left(t_{l}^{-}\right)-E_{X_{0}}\left(t_{l-1}^{-}\right)\;E_{X_{0}}\left(t_{l-1}^{+}\right)-{\left(E_{X_{0}}\left(t\right),\;E_{X_{0}}^{\prime }\left(t\right)\right)}_{l}-\frac{1}{\sigma }\;{\left({}_{0}I_{t}^{\left(1-\nu \right)}E_{X_{1}}\left(t\right),\;E_{X_{1}}\left(t\right)\right)}_{l}=0. \end{array}$$

To deal with the third term, we utilize the identity ${\left(u,\;\frac{du}{dt}\right)}_{l}=\left(u^{2}\left(t_{l}^{-}\right)-u^{2}\left(t_{l-1}^{+}\right)\right)/2$ with $u=E_{X_{0}}$. Hence, we multiply the preceding equation by two. Adding and subtracting $E_{X_{0}}^{2}\left(t_{l-1}^{-}\right)$ to the modified equation and rearranging the terms to obtain

$${\left(E_{X_{0}}\left(t_{l-1}^{+}\right)-E_{X_{0}}\left(t_{l-1}^{-}\right)\right)}^{2}+\left(E_{X_{0}}^{2}\left(t_{l}^{-}\right)-E_{X_{0}}^{2}\left(t_{l-1}^{-}\right)\right)-\frac{2}{\sigma }\;{\left({}_{0}I_{t}^{\left(1-\nu \right)}E_{X_{1}}\left(t\right),\;E_{X_{1}}\left(t\right)\right)}_{l}=0.$$

By summing over elements for l=1,…,J, we get

$$E_{X_{0}}^{2}\left(t_{J}^{-}\right)-E_{X_{0}}^{2}\left(t_{0}^{-}\right)+\sum_{l=1}^{J}{\left(E_{X_{0}}\left(t_{l-1}^{+}\right)-E_{X_{0}}\left(t_{l-1}^{-}\right)\right)}^{2}-\frac{2}{\sigma }\;{\langle {}_{0}I_{t}^{\left(1-\nu \right)}E_{X_{1}}\left(t\right),\;E_{X_{1}}\left(t\right)\rangle }_{J}=0.$$

By using Lemma 1, we have established the following stability of the LDG in the $L_{\infty }$ norm for (20) (see also References [38,40]:

**Lemma** **2.** *We have the following*$L_{\infty }$*stability of the LDG scheme* (20) *and for the numerical errors hold*

(22) $$E_{X_{0}}^{2}\left(t_{J}^{-}\right)=E_{X_{0}}^{2}\left(t_{0}^{-}\right)-\sum_{l=1}^{J}{\left(E_{X_{0}}\left(t_{l-1}^{+}\right)-E_{X_{0}}\left(t_{l-1}^{-}\right)\right)}^{2}+\frac{2}{\sigma }\cos\left(\frac{(1-\nu )\pi }{2}\right){∥E_{X_{1}}∥}_{H^{\frac{1-\nu }{2}}\left(\left[0,t_{J}\right]\right)}^{2}$$

We close this section by pointing out some facts about the order of convergence of the proposed LDG scheme. In Reference [38] it is shown that the solution can be calculated with optimal order of convergence (r+1) in the $L_{2}$ norm. In this work the mechanism of superconvergence is also discussed. The authors observed the superconvergence of order (r+1)+min{r,ν} at downwind point of each element.

## 5. Numerical Results and Discussions

In this section, we present some results of computations using the proposed LDG scheme described in the preceding sections to test their accuracy and efficiency when applied to the logistic equation. To assess the accuracy of the present numerical algorithms, we calculate the difference between the true exact and numerical solutions whenever the exact solution is available. For this purpose, we also consider a linear fractional population model and then we solve the fractional logistic equation numerically.

In order to asses the numerical scheme more qualitatively, by EOC we denote the estimated order of convergence calculated through defining

$$EOC:=\log_{2}\left(\frac{E_{a}\left(h\right)}{E_{a}\left(h/2\right)}\right),$$

where $E_{a}\left(h\right)$ is the absolute error corresponding to the step-size *h*. Moreover, to test the validity and accuracy of proposed LDG method and to make a comparison between our numerical model results with the results of other existing methods, we employ the predictor-corrector PECE method of Adams-Bashforth-Moulton type considered in Reference [43] as well as the implicit product integration of trapezoidal type described in Reference [24]. All experimental computations have been done by using MATLAB R2017a.

### 5.1. Linear Model

In this section, we consider a linear test problem to show the effectiveness of the proposed LDG approach. For this purpose, we consider the fractional population growth

(23) $$\left\{\begin{array}{cc} \; & {}_{a}^{\mathrm{LC}}D_{t}^{\nu }X\left(t\right)=\sigma^{\nu }\;X\left(t\right),\;t>0,\\ \; & X\left(0\right)=X_{0}, \end{array}\right.$$

where 0<ν≤1 and σ>0. This model problem is previously studied in Reference [22] and can be considered as a generalization of the Malthusian model (1) to the fractional-order derivative. By the aid of the Laplace transform, the exact analytical solution of the initial-value problem can be obtained in terms of well-knwon Mittag-Leffler function [10]

$$X\left(t\right)=X_{0}\;E_{\nu }\left(\sigma^{\nu }\;t^{\nu }\right),\;E_{\nu }\left(z\right)=\sum_{k=0}^{\infty }\frac{z^{k}}{\Gamma (k\;\nu +1)}.$$

Note that by taking ν=1 the exact solution becomes $X\left(t\right)=X_{0}\;e^{\sigma \;t}$.

To start computation, we take σ=1 for simplicity and set $X_{0}=3/4$. By considering ν=1 and J=1, the approximate solutions for r=3,6, and r=9 on the interval 0≤t≤2 are obtained as follows

$$\begin{array}{cc} Z_{0,3}\left(t\right) & =0.4233870968\;t^{3}-0.1814516129\;t^{2}+1.0887096774\;t+0.7016129032,\\ Z_{0,6}\left(t\right) & =0.003185535427\;t^{6}-0.00147024712\;t^{5}+0.04410741361\;t^{4}+0.1140555342\;t^{3}+0.3795910747\;t^{2}\\ \; & +0.7492022902\;t+0.7500339451,\\ Z_{0,9}\left(t\right) & =0.00000608710804\;t^{9}-0.00000288336716\;t^{8}+0.0002076022472\;t^{7}+0.0009466489455\;t^{6}\\ \; & +0.006344760802\;t^{5}+0.0311919123\;t^{4}+0.1250209298\;t^{3}+0.3749959904\;t^{2}+0.7500003306\;t\\ \; & +0.7499999933. \end{array}$$

**Figure 1:**
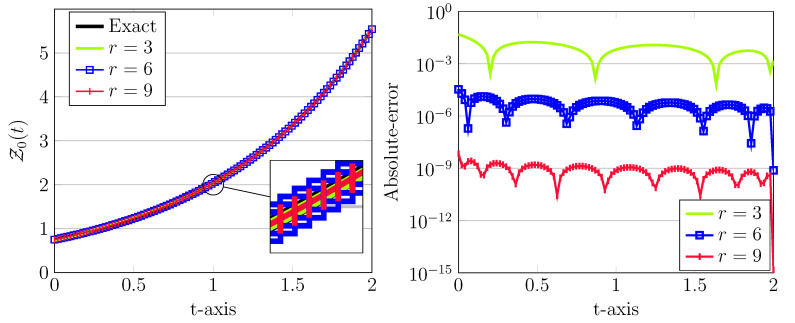
The approximated LDG with exact solutions (**left**) and the corresponding absolute errors (**right**) for J=1, ν=1, $\sigma =1,\;X_{0}=0.75$, and different r=3,6,9.

**Figure 2:**
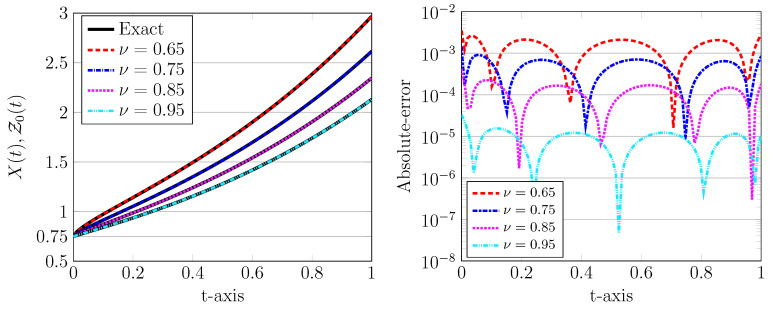
The approximated LDG with exact solutions (**left**) and the corresponding absolute errors (**right**) for J=1, r=5, $\sigma =1,X_{0}=0.75$, and various values of ν=0.65,0.75,0.85,0.95.

These approximations together with the corresponding absolute errors are depicted in Figure 1. Clearly, as *r* increased, more accurate results will be obtained. Note, in all cases, the step size is taken as h=2. Moreover, we emphasize that numerical solutions for this model problem based on the fractional spline collocation scheme have been proposed in Reference [22] with achieved absolute errors larger than $1\times 10^{-4}$, see Figure 2 in this paper. The parameters used in this approach related to ν=1 were $M_{1}=2^{6},2^{7},2^{8},N_{1}=37,69,133$, which obviously are much more greater than our used parameters.

Additionally, to justify our numerical model results, a comparison in Table 1 has been performed between the previous work on PECE [15,43] in terms of the number of (sub)intervals *J* is used in the computation. In this comparison, we compute the numerical solutions corresponding to X(2) as well as absolute errors $|X\left(2\right)-Z_{0}\left(2\right)|$ in these methods via different values of $J=2^{i}$ for i=0,1,…7. For our LDG method we take r=2 and ν=1. The last column in each method reports the corresponding EOC. The exact value of X(2) up to 30 digits is

X(2)=5.54179207419798736111715697916.

The observed EOC seen for PECE in Table 1 is approximately 2 as was proved in Reference [43]. However, the spuperconvergence EOC about 5 (≈2*r* + 1) is clearly achieved for our results. This comparison indicates the thoroughness of the proposed method.

The numerical solutions for various values of ν=0.65,0.75, 0.85,0.95 using r=5 and J=1 are depicted in Figure 2, left plot. In all plots, the exact solutions are indicated by a solid line while the numerical counterpart are visualized by (coloured) dotted, dashed, and dash-dotted curves. Note that the computational domain is [0,1], which implies that the time step is h=1. It can be seen from Figure 2 that the numerical solution obtained by the present LDG scheme has a good accuracy even using a relatively large time step and a low degree of the approximating polynomials. Furthermore, an appropriate choice of these computational parameters can improve the approximation accuracy.

Finally, for the linear model problem (23), we investigate the standard L1 approximation method [44] and its variant known as the fast L1 method [45]. To implement these approaches, we use a uniform mesh with the step size h=1/1000 on the interval [0,1]. In the LDG scheme, we utilize J=1 or h=1 and r=5 as the results shown in Figure 2. The numerical model results are presented in Table 2 for ν=0.75 and ν=0.5. For each ν, the corresponding exact solutions are also reported in the last column.

### 5.2. Nonlinear Model

We now consider the FLE (3) on [0,1] with the initial condition given by $X_{0}=1/2$ and the parameter σ=1/2. Using ν=1, the analytical exact solution of the logistic equation is given by

$$X\left(t\right)=\frac{1}{1+e^{-t/2}}.$$

**Figure 3:**
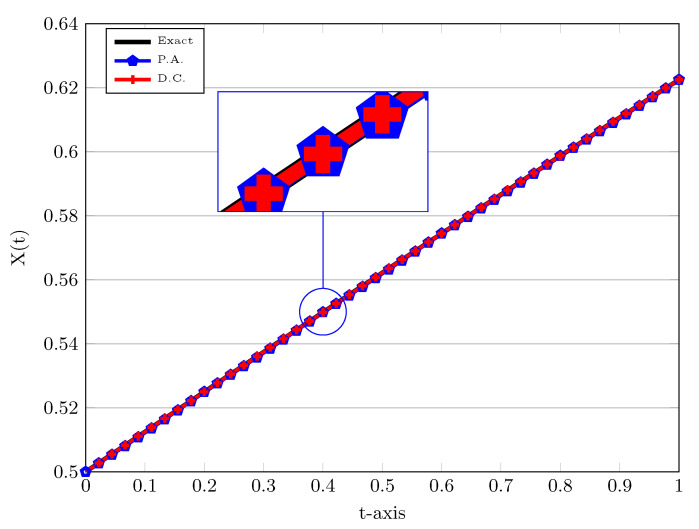
Numerical solutions of LDG scheme using P.A. and D.C. approaches with h=0.2, σ=0.5, $X_{0}=0.5$, and ν=1.0. The magnification of solutions at time t=0.4 is plotted in the box. The exact solution is displayed by a solid line.

**Figure 4:**
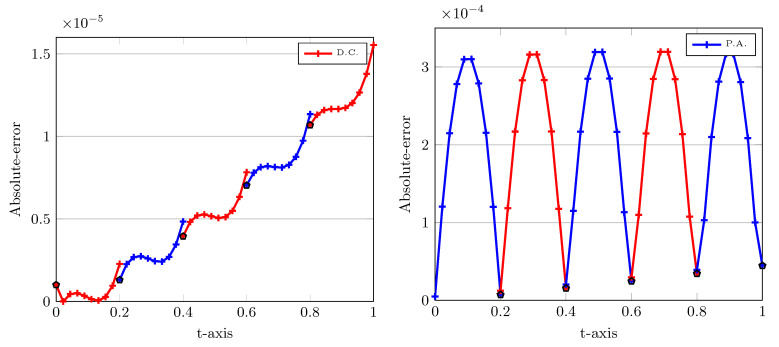
Absolute errors of LDG versus time using D.C. (**left**) and P.A. (**right**) approaches with h=0.2, σ=0.5, $X_{0}=0.5$, ν=1.0, and r=2. In the left and right plots, the upwind and downwind points are highlighted by black pentagon.

The simulation results for this example can be found in Figure 3 and Figure 4 for the number of elements equals to J=5 and the polynomial degree r=2. In Figure 3, we take ν=1 to compare the numerical results to the exact solution. Furthermore, we also use different approaches to treat the nonlinear term in the weak formulation, that is, the D.C. and P.A., which are utilized to compute $n_{j}^{DC}$ and $n_{i,j}^{PA}$ in (15). As one can see that from Figure 3 that a slightly more accurate result is obtained by means of direct computation rather than product approximation, however, as mentioned it is more time-consuming. In order to observe the behaviour of numerical solutions more closely, a magnification of these solutions at t=0.4 is done in Figure 3. The exact solution is depicted by a solid line.

In the next experiment, we plot the absolute errors when utilizing two approaches D.C. and P.A., as one observes in Figure 4. The computational parameters are the same as those applied for Figure 3. In Figure 4, the left plot corresponds to the D.C. and the right plot is when we use P.A. technique. Note that in all plots we have divided further each interval $L_{l}$ into ten subinterval uniformly to see the behaviour of the corresponding curves more precisely.

Let us interpret the numerical errors depicted in Figure 4. On the left picture in which the P.A. technique is used, the smallest errors are obtained at upwind points. Almost the same magnitude of errors is achieved at downwind points. On the contrary, on the right picture without using the P.A. this process is reversed. This implies that the minimum values of absolute errors are achieved at downwind points and there exist considerable difference between them and the errors obtained at upwind points in each $L_{l}$. In the next experiments, we compare the numerical errors achieved at the final point T=1.0, which is clearly a downwind point in the first approach.

In Table 3 and Table 4, we summarize the numerical results related to X(1) and its numerical approximation $Z_{0}\left(1\right)$ are obtained by the LDG procedure (9). Here, we use r=1,2 and a different choice of the number of grid points J=1,2,4,8 and 16 are utilized. In these tables, we further compare the performance of two different D.C. and P.A. approaches. All calculations are shown with 10 decimal places of accuracy. In the last column of each table, the estimated order of convergence (EOC) is given. The exact value is X(1)=0.622459331201855.

**Figure 5:**
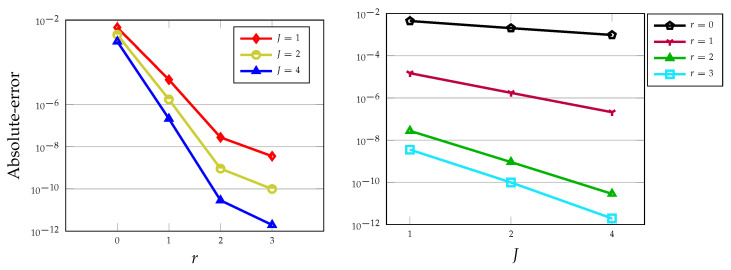
Absolute-errors versus polynomial degrees *r* for J=1,2,4 (**left**) and against the number of elements *J* for r=0,1,2,3 (**right**) evaluated at T=1.0 and for ν=1.

It can be seen from Table 3 and Table 4 that using r=1 and r=2 in the D.C. approach, the results are accurate respectively to 6 and 10 decimal places for only J=4 intervals. In other words, achieving an order of accuracy equal to 3 and 5 is possible if one uses the LDG scheme with r=1,2 degree of polynomials and for a small number of elements. These EOC are also confirmed the superconvergence order at downwind points previously reported in Reference [38]. Note that by utilizing the P.A. technique, the obtained EOC is equal to 2. We emphasize also that using the scheme PECE for the nonlinear logistic equation the EOC at most 2 will be achieved and of course a larger number of intervals *J* is required. In the next plot, we examine the behaviour of the absolute errors in the log scale for various polynomial degrees as well as with respect to the number of elements *J*, see Figure 5.

**Figure 6:**
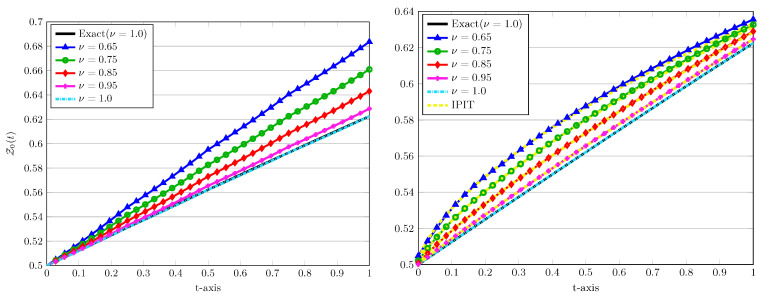
The approximated LDG solutions versus time using P.A. (**left**) and D.C. (**right**) approaches with J=4, r=3, $\sigma ,X_{0}=0.5$, and various values of ν=0.65,0.75,0.85,0.95,1.0.

In the next experiment we show the impact of the fractional derivative on the approximated obtained solutions. In Figure 6 we present the approximated solutions at J=4, r=3 with different values of the fractional derivatives ν=0.65,0.75,0.85,0.95 as well as ν=1.0. In these plots, we also compare the performance of two P.A. and D.C. approaches for these values of ν. In each case, for ν=1.0 the exact solution is also shown by a solid line. To justify our computed results, the implicit product-integration of trapezoidal (IPIT) rule with the step size h=1/256 is used [24].

From both depictions in Figure 6, one can observe that the numerical solutions for ν∈(0,1) are approaching to the solutions correspond to ν=1 for which the exact solution is known. Of course, more reliable results is obtained through the D.C. as previously tested for ν=1 in Table 3 and Table 4.

## 6. Conclusions

In this work, an approximation algorithm based on the LDG scheme is developed for the fractional-order logistic equation occurring in many biological and chemical phenomena. To be more precise, our numerical scheme based on discontinuous Galerkin finite element concept with Legendre basis functions yields to a set of nonlinear equations to be solved in each subinterval. The numerical stability in the linear case is proved and the order of convergence is also discussed. Beside the direct computation of the nonlinear term, the technique of product approximation is also utilized and then their performance are compared for various J,r and ν. We have tested the performance of the LDG scheme on the linear as well as nonlinear growth and logistic differential equations of fractional order. Comparing our numerical results with the PECE indicates that the present approaches produce an accurate approximation for the underlying model problems.

## Figures and Tables

**Table 1:** Comparison of absolute errors in LDG with r=2 and PECE for different number of interval *J* and ν=1. Numbers in bold show that the correct digits are obtained by the LDG.

	LDG			PECE		
J	$Z_{0}\left(2\right)$	$|X\left(2\right)-Z_{0}\left(2\right)|$	**EOC**	**Numerical**	**Error**	**EOC**
1	**5.625000000000**	$8.3208_{-2}$	−	3.750000000000	$1.7918_{+0}$	−
2	**5.543701171875**	$1.9091_{-3}$	5.45	4.687500000000	$0.8543_{+0}$	1.07
4	5.541845071676	$5.2998_{-5}$	5.17	**5**.229675292969	$0.3121_{+0}$	1.45
8	5.541793647744	$1.5735_{-6}$	5.07	**5**.446685392454	$9.5107_{-2}$	1.71
16	5.541792122228	$4.8030_{-8}$	5.03	5.515562177333	$2.6230_{-2}$	1.86
32	5.541792075682	$1.4842_{-9}$	5.02	5.534910274764	$6.8817_{-3}$	1.93
64	5.541792074244	$4.6126_{-11}$	5.01	5.540030137766	$1.7619_{-3}$	1.97
128	5.541792074199	$1.4380_{-12}$	5.00	5.541346351966	$4.4572_{-4}$	1.98

**Table 2:** Comparison of numerical solutions in LDG with r=5, h=1 and L1/fast L1 schemes with $h=10^{-3}$ for some t∈[0,1] and ν=0.75,0.5.

	ν=0.75				ν=0.5			
t	**LDG**	**L1**	**Fast L1**	**Exact**	**LDG**	**L1**	**Fast L1**	**Exact**
0.2	1.0536	1.0524	1.0524	1.053507	1.3420	1.3459	1.3345	1.349263
0.4	1.3512	1.3486	1.3486	1.350342	1.8370	1.8176	1.8176	1.822532
0.6	1.6963	1.6945	1.6945	1.697186	2.3489	2.3525	2.3525	2.359660
0.8	2.1128	2.1087	2.1087	2.112499	2.9957	2.9845	2.9845	2.994627
1.0	2.6134	2.6091	2.6091	2.614400	3.7385	3.7427	3.7427	3.756735

**Table 3:** Comparison of absolute errors in LDG with r=1 using P.A. and D.C. for different number of interval *J* and ν=1. Numbers in bold show that the correct digits are obtained by the LDG.

	P.A.			D.C.		
J	$Z_{0}\left(1\right)$	$|X\left(1\right)-Z_{0}\left(1\right)|$	**EOC**	$Z_{0}\left(1\right)$	$|X\left(1\right)-Z_{0}\left(1\right)|$	**EOC**
1	**0.6234038976**	$0.9445664060_{-3}$	−	0.6224742460	$0.1491482269_{-4}$	−
2	**0.6226973939**	$0.2380627190_{-3}$	1.99	0.6224610781	$0.1746857403_{-5}$	3.09
4	0.6225290166	$0.6968541429_{-4}$	1.77	0.6224595421	$0.2108842001_{-6}$	3.05

**Table 4:** Comparison of absolute errors in LDG with r=2 using P.A. and D.C. for different number of interval *J* and ν=1. Numbers in bold show that the correct digits are obtained by the LDG.

	P.A.			D.C.		
J	$Z_{0}\left(1\right)$	$|X\left(1\right)-Z_{0}\left(1\right)|$	**EOC**	$Z_{0}\left(1\right)$	$|X\left(1\right)-Z_{0}\left(1\right)|$	**EOC**
1	**0.6233820141**	$0.9226828763_{-3}$	−	0.6224593588	$0.2759267670_{-7}$	−
2	**0.6226943815**	$0.2350503824_{-3}$	1.97	0.6224593321	$0.9149985214_{-9}$	4.91
4	0.6225286311	$0.6929984936_{-4}$	1.76	0.6224593312	$0.2863453918_{-10}$	5.00
